# Joint modeling of blood pressure measurement and survival time of hypertension patients

**DOI:** 10.1038/s41598-021-94905-y

**Published:** 2021-08-03

**Authors:** Hakime Ayele Kosa, Markos Abiso Erango

**Affiliations:** Department of Statistics, Collage of Natural Science, Arba Minch University, Arba Minch, Ethiopia

**Keywords:** Health care, Disease prevention

## Abstract

Hypertension is a chronic disease that has a major health problem over the centuries due to its significant contribution to the global burden. The objective of this study was to examine the association of survival time and longitudinal Systolic Blood Pressure (SBP) measurement and finding potential barrier that affects SBP measurement and the survival time of hypertension patients. The study considered a cohort of 318 hypertension patients who were aged 18 years or older and were under follow-up from January 1, 2012, to February 30, 2020, at Arba Minch General Hospital. To analyze the data we employed linear mixed effect model, Weibull model, and joint modeling approach for longitudinal and survival data. The results from joint model analysis indicate that systolic blood pressure measurement is significantly associated with survival time of hypertension patients. The results from the longitudinal sub-model reveals that alcohol use, Khat intake, smoking tobacco, stages of hypertension diseases, adherence to treatment, related diseases, and family history had statistical significant relationship with mean change in the $\sqrt{SBP}$ measurement. Furthermore, from the survival sub-model, we found the survival probability of hypertension patients was determined by family history, stages of hypertension disease, related diseases, and smoking tobacco. The analysis suggests that there is a strong association between SBP measurement and survival time of hypertension patients. Thus we recommend aggressive work by all concerned bodies towards awareness creation about the effect of potential barriers.

## Introduction

Hypertension is one of major worldwide public health challenge and it is root cause of many body system and organs failure in both developed and developing counties. In 2008 Overall prevalence of hypertension in adult age 25 and above were 40% and it was cause for 7.5 million deaths worldwide^1–3^. Recent researches indicate that 972 million people living with hypertension in year 2000 in the globe, and it will be increased more than 1.56 million in the year 2025^4^. Globally the number of people with uncontrolled hypertension rose by 70% between in the year 1980 and 2008; the rising epidemic of hypertension is due to modernization, population growth and ageing^5^.

Hypertension in sub-Saharan Africa is widespread of immense economic importance because of its high prevalence in urban areas, its frequency under diagnosis and the severity of its complications. Now a day it is transferred from a comparative rarity to the most important public health problem and cardiovascular disease is a unique double burden challenge for the whole of Africa^6^. The estimated number of hypertensive patients in Sub-Saharan Africa in 2025 will be 150 million^7^. Additionally, there is evidence which shows complications of hypertension such as stroke and heart failure are increasing at an alarming rate in this region^8^. Current disease assessments for Sub-Saharan Africa indicate that there is a wide disproportion (0.4–43%) in the prevalence of hypertension and obesity. The detection rates in most high-income countries vary from 32 to 64% although in many low-income countries, the reported detection rates are considerably lower^9^.

In Ethiopia, due to economic development and rapid growth of urbanization, non-communicable diseases and their related risk factors are growing and becoming a double burden^10^. The prevention and control of hypertension has not given attention in Ethiopia compared with other diseases like HIV/AIDS, Tuberculosis, malaria and nutritional deficiency diseases^11^.

In clinical and medical studies, longitudinal and survival data are considered important measures of health, and most of the time they arise together^12^. Longitudinal observations such as square root of SBP are obtained from repeated measurements on the hypertensive patients at a series of time points. The related data on time-to-death event describe the length of time for occurrence of the event on each individual patient during a specified study period. So, Joint models analyses of such kind of data’s important to incorporate all information simultaneously and provide effective and efficient inferences^13^. The goal of this study was to examine the association of survival time and longitudinal SBP measurement and finding potential barrier that affects SBP measurement and the survival time of hypertension patients.

## Methodology

### Data descriptions

The data for this study collected from hypertension patients under follow up at Arba Minch general hospital located in SNNPR Gamo zone Arba Minch town Ethiopia from January 1, 2012 to February 30, 2020. The data were taken out from the patient’s registration chart and cards with epidemiological, laboratory and clinical information of the patients under follow-up. Among 1020 hypertension patients under follow up 318 patients was selected with simple random sampling technique and the data was analyzed using R software version 3.6.2. The research proposal of this study was checked and approved by ethical clearance committee of Arba Minch University. So, Informed consent has been waived by Arba Minch University of the ethical committee. All study procedures were performed following relevant guidelines and regulations laid down by the Committee.

### Ethics declarations

The letter of ethical clearance was obtained from institutional review board of Arba Minch University, and Arba Minch General hospital gave permission to collect data of patients from recorded cards. For the purpose of confidentiality, there were no links with individual patients and all data had no personal identifier and were kept confidential and therefore Informed consent has been waived by Arba Minch University ethical committee.

### Study variables

Measurements of systolic blood pressure and survival time of hypertension patients were considered as response variable in this study. Systolic blood pressure measurement was measured repeatedly over time for each hypertension patient under the follow up. SBP has been a better indicator of risk than DBP^14^; it measured at least three Blood Pressure measurements after the first report of hypertension diagnosis and the survival time of patients was the time in months of a patient to the associated death event. And it is time to death event of the patient under the follow up.

The predictor variables assumed to be determined the longitudinal systolic blood pressure (SBP) measurement and survival time of hypertension patients were visit time, Gender (Female, Male), Base line age $(\le 50,>50$), place of residence (Rural, Urban), alcohol use (No, Yes), Khat intake (No, Yes), smoking tobacco (No, Yes), status of stress (No, Yes), stages of hypertension (Normal, Elevated, Stage1, Stage2 and Hypertensive crisis) disease, life style change (Yes, No), blood cholesterol level(Normal, Raised), adherence to treatment(Poor, Good), Related diseases (None, Stroke, Heart case and Others), family history of hypertension patients (Positive, Negative), Diabetes of mellitus status (No, Yes),and Base line fasting blood sugar (8 h fasting measure of blood sugar (Continuous) were considered**.**

### Methods of data analysis

The analysis consists; linear mixed effects model for the longitudinal data, Weibull regression model for the time to event data and joint model for both longitudinal and time to event data.

#### Linear mixed effects model

In longitudinal data analysis, variations occurred from two sources, within-subject and between subject, within-subject arises during the measurements within each subject, and between subject variations arises during the measurement between different subjects. This variation among individuals arises because of unmeasured factors^15^. Each person in the population is supposed to have his/her own mean response trajectories.

A linear mixed model contains fixed and random effects. The fixed effect section of the model represents the mean response whereas the random effects section represents the individual level responses. The general linear mixed effect model for the longitudinal data defined as^16^:

1 $${Y}_{i}={X}_{i}\beta +{Z}_{i}{u}_{i}+{\varepsilon }_{i},$$

$$\left\{\begin{array}{c}{u}_{i}\sim N(0,D)\\ {\varepsilon }_{i}\sim N(0,\Sigma )\\ {u}_{i},\dots ,{u}_{n} \, and \, {\varepsilon }_{1},\dots ,{\varepsilon }_{n} \; are \; independent,\end{array}\right.$$

where, ${Y}_{i} \mathrm{is}$ ${n}_{i}\times 1$ response vector of the *i*th subjects, *ε*_*i*_ is distributed as $N(0,\Sigma )$ is a vector of residuals components,${u}_{i}$ is distributed as $N(0,\Omega )$, independently of each other.

#### Covariance structures

A model for the covariance must be chosen on the basis of some assumed model for the mean response. In order to reduce the number of parameters in the variance–covariance structure $\Sigma$, we can fit models with more structures that are parsimonious. The following are commonly used variance covariance structure ($\Sigma$) among others: Independent (IND), Compound symmetry (CS), Heterogeneous compound symmetry (CSH), and Unstructured (UN). These often lead to more efficient inferences for the mean parameters and particularly useful when many repeated measurements are taken per subject^17^.

#### Random intercept model

The random effects model or subject-specific model assumes that extra correlation arises among longitudinal response^15^. The random intercepts model allows intercepts to vary across groups. In particular, a basic example of a random intercepts model which is included in order to illustrate the model fitting is formed by two clearly distinct parts,

2 $${Y}_{i}={{\beta }_{0}+{\beta }_{1}X}_{ij}+{u}_{0i}+{\varepsilon }_{i}.$$

These are, a fixed part (which is the intercept and the coefficient of the explanatory variable times the explanatory variable) and a random part. The random effect *u*_*i*_ and within-subject error *ε*_*i*_ are independent for different subjects and independent of each other for the same subject. i.e.

$$cov\left({\varepsilon }_{i},{\varepsilon }_{j}\right)=0, if \, i\ne j, cov\left({u}_{i},{u}_{j}\right)=0,if \, i\ne j, \; \mathrm{and} \; cov\left({u}_{i},{\varepsilon }_{j}\right)=0.$$

In the mixed model formulation, the design matrices are replaced by:

$${X}_{i}=\left[\begin{array}{cc}{1}_{1}& {x}_{i1}\\ \vdots & \vdots \\ {1}_{ni}& {x}_{ini}\end{array}\right], \, {Z}_{i}=\left[\begin{array}{c}{1}_{1}\\ \vdots \\ {x}_{ini}\end{array}\right], \; \beta ={\left[\left.{\beta }_{0},{\beta }_{1}\right]\right.}^{T}.$$

And the random effects model covariance structure, ${u}_{i}\sim N(0,D)$ with ${D}_{i}={{\delta }_{u}}^{2}.$

#### Random intercept and random slope model

An intuitive extension that allows a random shift in the subject-specific slopes is known as random intercepts and random slopes model. Consider the simple random intercepts and slopes model^15^,

3 $${Y}_{i}={{\beta }_{0}+{\beta }_{1}X}_{ij}+{u}_{0i}+{u}_{1i}{t}_{ij}+{\varepsilon }_{i}.$$

In this model, we have additional *u*_1*i*_ which represents the random slope effect of the coefficients and ${X}_{ij}=j=\mathrm{1,2},\dots {n}_{j}$, of *j*th response on *i*th subject. As a result, actually two extra parameters should be estimated: the variance in intercepts between groups ${{\delta }_{u0}}^{2}$ and the variance in slopes between groups ${{\delta }_{u1}}^{2}$. In this case random effect design matrix ${Z}_{i}$ has the form,

$${Z}_{i}= \left \lceil\begin{array}{c}{1}_{1} \, {x}_{n}\\ \vdots \vdots \\ {1}_{ni} \, {x}_{nn}\end{array} \right \rceil \; \mathrm{and \; the \; random \; effects \; model \; covariance \; structure},$$

$$\left[\begin{array}{c}{u}_{0i}\\ {u}_{1i}\end{array}\right]\sim N\left(0,D\right) \; with \; {D}_{i}=\left[\begin{array}{cc}{\delta }_{u0}^{2}& {\delta }_{u0u1}^{2}\\ {\delta }_{u0u1}^{2}& {\delta }_{u1}^{2}\end{array}\right],$$

where ${\delta }_{u0ui}$ denotes the covariance between the intercepts and slopes.

#### Weibull regression model

The most important characteristic that distinguishes the analysis of survival times from other areas in statistics is censoring. Subjects are said to be censored if they are lost to follow up, withdrawing from the study, or if the study ends before they have an outcome of interest.

That is, observations are called censored when information about their survival time is incomplete. There are four kinds of censoring: right censoring, left censoring, interval censoring, and truncation^18^. By far the most common type of censoring is right censoring, which occurs when observation is terminated before an individual experiences the event of interest. This could happen if a patient survives through the experiment and was still alive when the experiment ends.

The Weibull regression model is employed frequently in modeling survival data. The Weibull regression model commonly used to handle survival data with monotonic hazard behavior. But, when a hazard function is non-monotonic, log-logistic and lognormal distributions are useful alternatives. Log-logistic and lognormal distributions have hazard functions that each reaches a peak and then decline over a period of time^19^

In this study, we assume that the survival time for the *i*th subject follows a Weibull distribution. Suppose the survival time *T* has Weibull distribution with scale parameter λ_i_ (t) and shape parameter ρ. Then under AFT model, the survival time T_i_ of the *i*th individual is distributed as

4 $${\mathrm{T}}_{i} \sim \mathrm{ Weibull } \, \left(\uprho ,\mathrm{ \lambda i }\left(\mathrm{t}\right)\right),$$

where $$\mathrm{log }\left(\mathrm{\lambda i }\left(\mathrm{t}\right)\right)={X}_{2i}^{T}\left(t\right)\alpha .$$

### Joint longitudinal: survival models

A use of joint models, which gains increasing interest in recent years, refers to the statistical analysis of the resulting data while taking account of any association between the repeated measurement and time-to-event outcomes^20^. Joint longitudinal-survival models can be formed where the association between the two endpoints is due to shared random effects.

In joint model, the longitudinal and survival processes are assumed to be conditionally independent given unobserved random effects. That is, the key assumption of a joint model is that the random effects underlie both the longitudinal and survival processes. This means that these random effects account for both the association between the longitudinal and time to event outcomes, and the correlation between the repeated measurements in the longitudinal process. This type of joint model is also called a shared parameter model, as both processes share these random effects^21^. The joint model consists of two linked sub-models, known as the longitudinal sub-model, and the survival sub-model.

#### Longitudinal sub-model

We assume that the hazard function ${\lambda }_{i}(\mathrm{t})$ depends on the true longitudinal outcome ${m}_{i}(\mathrm{t})$ at time *t*. However, for each subject, we may have this longitudinal outcome occasionally at times $\left\{{t}_{ij},j=\mathrm{1,2},\dots {m}_{i}\right\}$ with measurement errors. Therefore, to examine the impact of the longitudinal outcome to the hazard for an event, we need to estimate ${m}_{i}(\mathrm{t})$ for each individual. We can accomplish this by fitting a mixed effects model with the available longitudinal measurements ${y}_{ij}=\left\{{y}_{ij}\left({t}_{ij}\right),j=\mathrm{1,2},\dots {m}_{i}\right\}$ of *i*th subject. Normally distributed longitudinal outcomes and linear mixed effects (LME) model^22^ can be given by:

$$\begin{aligned} y_{i} \left( t \right) & = X^{\prime}_{i} \left( t \right)\beta + Z_{i}^{^{\prime}} u_{i} + \varepsilon_{i} \left( t \right) \\ & = m_{i} \left( t \right) + \varepsilon_{i} \left( t \right), \\ \end{aligned}$$

5 $${m}_{i}\left(t\right)={{X}^{^{\prime}}}_{i}\left(t\right)\beta +{Z}_{i}^{^{\prime}}{u}_{i},{u}_{i}\sim N\left(0,G\right) \; and \; {\varepsilon }_{i}\left(t\right)\sim N\left(0,{\delta }^{2}\right).$$

#### The survival sub-model

The survival model in the joint model includes a latent association function ${W}_{2i}(t)$, thus, the survival sub- model is defined as:

$$Log\left(T/{W}_{2i}\right)={{X}_{2i}}^{T}(\mathrm{t})\alpha + {W}_{2i}\left({t}_{ij}\right)+{\in }_{{\varvec{i}}{\varvec{j}}},$$

where, $\alpha$ is a vector of unknown and fixed coefficient of the covariates,${W}_{2i}($t)refers to subject specific random effects of the survival time having Gaussian distribution, ${\in }_{{\varvec{i}}}$ is a sequence mutually independent measurement errors that follows AFT distribution. There are many ways of making the linkages between longitudinal and survival sub models. In this study we consider the links used in Ref.^23^.

#### Estimation methods

In general terms, we use efficient estimation method using likelihood based models either ML or REML estimation to obtain estimates of the covariance parameters in linear mixed models with the remark that REML is usually better than ML, because it reduces the well-known finite sample bias in the estimation of the covariance^24^. The distinction between ML and REML is the construction of the likelihood function. However, the two methods are asymptotically equivalent and often give very similar results except the difference becomes important only when the number of fixed effects is relatively large.

In order to estimate the survival function, the parameter estimation and their estimated variances in the Weibull regression model can be found by maximizing the log-partial likelihood function with respect to the parameters. Let the sub-index *i* refer to the individual indicator and consequently, $\left\{,{X}_{i}{,\delta }_{i}\right\},i=\mathrm{1,2},\dots n$ denote their survival information. Taking a random sample from a certain distribution, parameterized by *θ*, the likelihood function is given by,

7 $$l\left(\theta \right)=\prod f({x}_{i},{\theta )}^{{\delta }_{i}}{S}_{i}({x}_{i},{\theta )}^{{1-\delta }_{i}}.$$

Note that it takes to account for censoring information, by contributing with $f({T}_{i},\theta )$ when an event is observed at time ${T}_{i}$ and with $f({T}_{i},\theta )$ when subjects survived up to that point, that is ${T}_{i}>{X}_{i}={C}_{i}$. This can be rewritten in terms of hazard function as,

8 $$l\left(\theta \right)=\prod \lambda ({x}_{i},{\theta )}^{{\delta }_{i}} exp(-\lambda (t{)}^{{1-\delta }_{i}},$$

where $\lambda (\cdot )$ is the cumulative risk function, which describes the probability that the event of interest has occurred up until time, To address this issue, iterative optimization procedures could be necessary to locate the maximum likelihood estimates $\widehat{\theta }$ using iterative numerical analysis techniques often done via the Newton–Raphson algorithm^25^, which is based on the following iterative procedure: ${\widehat{\beta }}_{new}={\widehat{\beta }}_{old}+{I}^{-1}({\widehat{\beta }}_{old})U({\widehat{\beta }}_{old})$ with $U({\widehat{\beta }}_{old})$ is the vector of scores and ${I}^{-1}({\widehat{\beta }}_{old})$ is the inverse of the observed information matrix. The Convergence is reached when ${\widehat{\beta }}_{old}$ and ${\widehat{\beta }}_{new}$ is sufficiently close together.

The main estimation method that has been proposed for joint models is ML. The standard ML method involves maximizing the log-likelihood, corresponding to the joint distribution of the time-to-event and longitudinal data processes. Strictly, both processes share the same unobserved random effects, and are conditionally independent given these random effects^22^, thus

9 $$\begin{aligned} & f\left( {T_{i} ,\delta_{i} ,y_{i} {|}u_{i} ,\theta } \right) = f\left( {T_{i} ,\delta_{i} {|}u_{i} ,\theta } \right)f\left( {y_{i} {|}u_{i} ,\theta } \right) \\ & {\text{with }}\,f\left( {y_{i} {|}u_{i} ,\theta } \right) = \prod f\left( {y_{i} \left( {t_{ij} } \right){|}u_{i} ,\theta } \right). \\ \end{aligned}$$

Because of the fact that the survival and longitudinal sub-models share the same random effects, joint models of this type are also known as shared random effects models. Under these conditional independence assumptions between longitudinal outcome and time-to-event given the random effects ${u}_{i}$, the joint log-likelihood contribution of the *i*′th subject is expressed as

10 $$\begin{aligned} Log \, f\left( {T_{i} ,\delta_{i} ,y_{i} ,\theta } \right) & = log\int {f\left( {T_{i} ,\delta_{i} ,y_{i} ,\theta } \right)du_{i} } \\ & = log\int {f\left( {T_{i} ,\delta_{i} {|}u_{i} ,\theta_{t} ,\beta } \right)\left[ {\prod f\left( {y_{i} \left( {t_{ij} } \right){|}u_{i} ,\theta_{y} } \right)} \right]f\left( {u_{i} ,\theta_{b} } \right)du_{i} } , \\ \end{aligned}$$

where ${\theta }_{t},{\theta }_{y}$, and ${\theta }_{u}$ represent the parameters for the survival process, the longitudinal process and the random effects respectively, $f\left({y}_{i}({t}_{ij})|{u}_{i},{\theta }_{y}\right)$ is the density for the longitudinal process and $f({u}_{i},{\theta }_{u})$ is the density for the random effects. The likelihood of the survival part $f\left(({T}_{i},{\delta }_{i}|{u}_{i},{\theta }_{t},\beta \right)$ is written as,

11 $$f\left(({T}_{i},{\delta }_{i}|{u}_{i},{\theta }_{t},\beta \right)=[\left({\lambda }_{i}({T}_{i}|{M}_{i}({T}_{i}\right),{\theta }_{t},\beta {]}^{{\delta }_{i}} S(\left({T}_{i}|{M}_{i}\left({T}_{i}\right),{\theta }_{t},\beta \right).$$

And, the survivor function for the *i*′th subjects is given by,

12 $$\begin{aligned} S\left( {t{|}M_{i} \left( t \right),\omega_{i} ,\theta_{t} ,\beta } \right) & = pr(T_{i} > t\left( {M_{i} \left( t \right),\omega_{i} ,\theta_{t} ,\beta } \right) \\ & = exp\left\{ { - \mathop \smallint \limits_{0}^{1} \lambda_{i} \left( {s{|}M_{i} \left( s \right),\theta_{t} ,\beta } \right)dus} \right\}. \\ \end{aligned}$$

The log-likelihood for the joint model is approximated using the Expectation–Maximization (EM) algorithm, because both the integral with respect to the random effects and survival function typically do not have an analytical solution, except in some special cases.

### Ethical consideration

The research proposal of this study was checked and approved by ethical clearance committee of Arba Minch University and data collected from Arba Minch General Hospital recorded cards, and Arba Minch General Hospital medical directors gave permission to use the patient’s data for this study. For the purpose of confidentiality, there were no links with individual patients and all data had no personal identifier and were kept confidential.

## Results

### Descriptive analysis

From the total sample population, major parts (54.4%) were females and the remaining (45.6%) were males. Among those patients considered in the study we observed that (11%) died while the remaining (89%) were censored. The death proportion of female patients was (11%) which is greater than male patients are (7.58%). Regarding place of residence about 64.78% of patients were living in urban areas and 35.22% of them in rural areas with death proportion of 11.66%, 6.87% respectively. Regarding age proportion, the majority of the hypertension patients were at age of above 50 was 51.13% which is greater than the age group below 50 which is 48.87%.The highest death proportion was observed from patients who use alcohol (16.176%) whereas the lowest proportion of death (6.87%) was observed among a patient who does not use alcohol.

The proportion of death was varied by Khat intake of the patients. The highest proportion of death was observed from a patient who intake Khat (23.59%) whereas the lowest death proportion (5.28%) was recorded among patients who do not intake Khat. Regarding blood cholesterol level, Patients who have raised blood cholesterol level have highest proportion of death (22.43%) than patients those who have normal blood cholesterol level. In addition, patients who have practicing modification of life style change have low death proportion (8.84%) than patients those who have not practicing modification life style change (12.38%).

Regarding hypertension disease stages those who was in the normal stage, elevated stage, stage_1,_ stage_2_ and hypertensive crisis stage were (8.2%), (14.41%), (24.6%), (32.8%) and (20%) respectively; the risks of death proportion were increasing as compared to normal stages.

The risk of dying proportions of patients who have stroke, heart case, others and none related diseases were 30.95%,17.89%, 4.082% and 1.78% respectively. The highest part of dying was recorded from them with poor adherence (11.66%) whereas the lowest part of death (6.87%) was accounted among sample population with good adherence.

In addition, (64.46%) patients had positive family history of hypertension disease and the remaining (35.53%) had negative family history of hypertension case, and their death proportion was 11.255%, 7.317%respectively. From the total sample subjects (61.32%) of them had diabetes mellitus case and the remaining (38.68%) of them had not diabetes mellitus disease.

### Analysis for survival time

The patients were following up for the total 65 months. The median survival time of hypertensive patients was 38 months with mean and standard deviation of 37.74 and 19.97 months, respectively.

**Figure 1:**
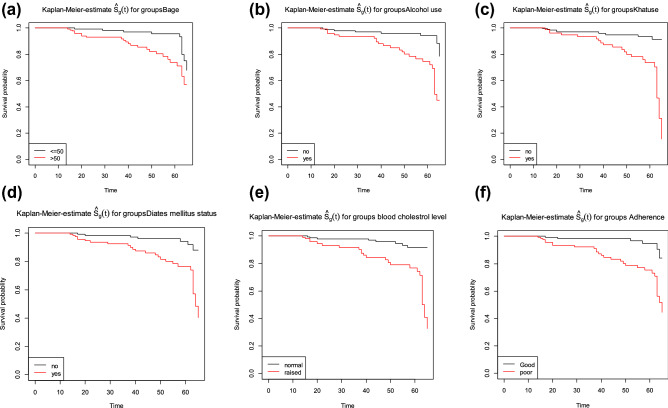
Plot of Kaplan–Meier survival function curves of hypertension patients (**a**) Age (**b**) Alcohol (**c**) Khat (**d**) diabetes Mellitus status (**e**) Cholesterol level (**f**) Adherence.

Comparison of survival function is a good indication to see the event experiencing time of the groups. The estimation of survival function graphs in Fig. 1 indicates the decreasing pattern of survival ship graphs as expected. The Kaplan–Meier life span plots showed the pattern of one survival function lying above another, indicating the group defined by the upper curve had a better survival probability or life span probability than the group defined by the lower curve.

Looking in to survival function of base line age; less than and 50 had higher experience of lifespan probability than baseline age greater than 50.When compare subjects with alcohol use, the patients who do not use alcohol had better survival probability than those who use alcohol. Comparing the lifespan function of the sample population with cholesterol level, subjects who have the normal blood cholesterol level had higher experience of survival probability or lifespan than those who have raised blood cholesterol level. Also the survival function of patients taking Khat was lower survival probability or lifespan than patients does not taking Khat. In addition, subjects who have positive family history of hypertension disease had less experience of survival probability compare to patients with negative family history of hypertension cases. The patients who had good adherence have more favorable survival experience than patient who had poor adherence and they who have not diabetes of mellitus case had better survival experience than patients who have diabetes mellitus.

The log rank test was used to check the significance of observed difference among categories of covariates using chi-square. The rank test results showed that there were significance differences in survival probability of patients in different categories of age (*χ*^2^ = 2.3, p < 0.002), alcohol use (*χ*^2^ = 5.7, p < 0.02), Khatintake (*χ*^2^ = 6.6, p < 0.01), smoking tobacco (*χ*^2^ = 24.1, p < 0.00), blood cholesterol level (*χ*^2^ = 4.3, p < 0.04), adherence to treatment (*χ*^2^ = 5.8, p < 0.02), related diseases (*χ*^2^ = 17.5, p < 0.008), Status of stress (*χ*^2^ = 11.6, p ≤ 0.00),and family history of hypertension patients(*χ*^2^ = 3.8, p < 0.005) and diabetes mellitus status(*χ*^2^ = 6.7,* p* < 0.01) at 5% level of significance. However, there was no significance difference in survival probability between the categories of gender, place of residence, stages of hypertension diseases and lifestyle change (modification) of hypertensive patients.

### Exploring individual profiles

**Figure 2:**
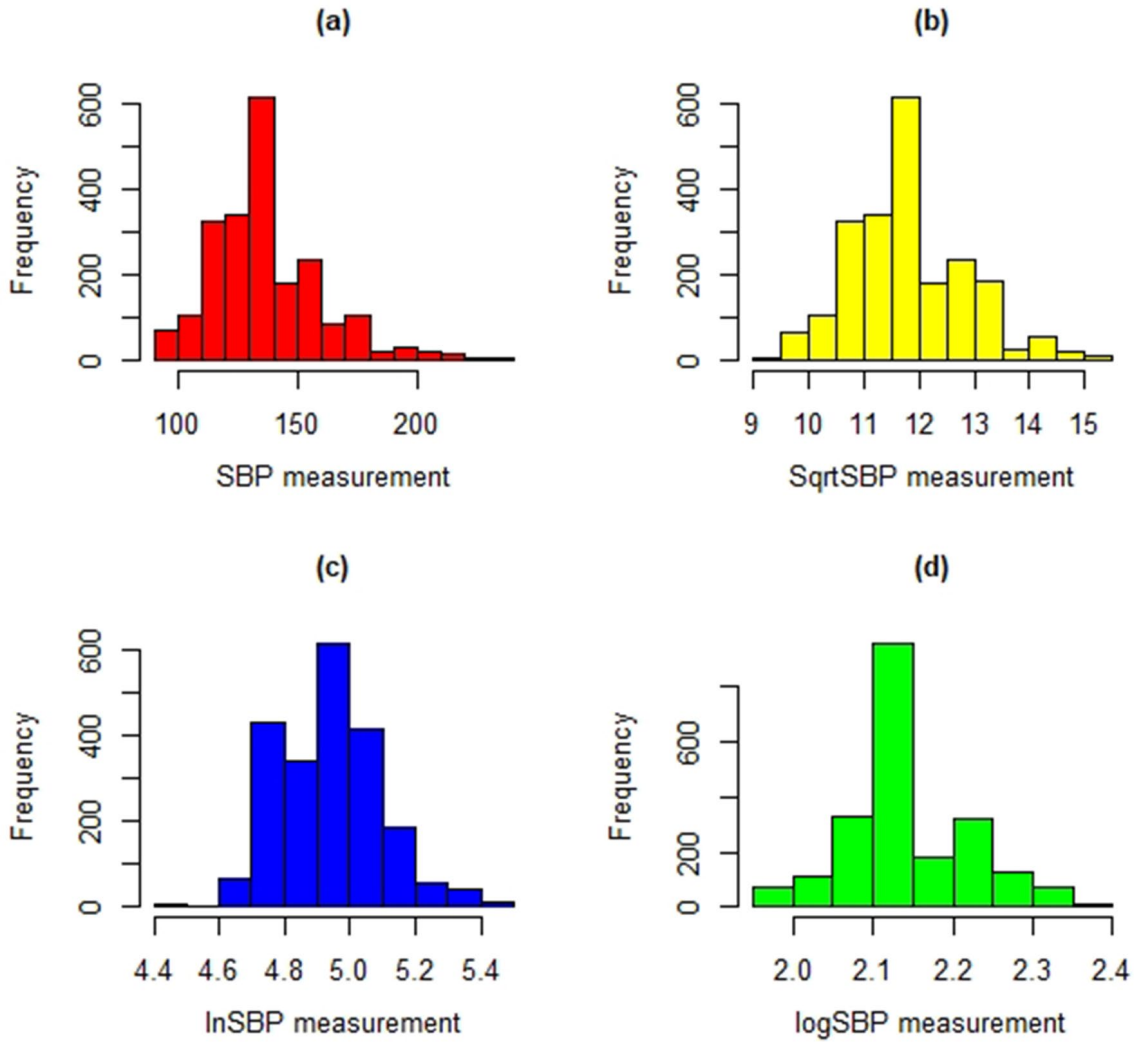
Histogram of the SBP measurement (**a**) SBP (**b**) Square root of SBP (**c**) Natural logarithms of SBP (**d**) logarithms of SBP.

In longitudinal data analysis, histograms and normal Q–Q plots of the systolic blood pressure (SBP) measurements with corresponding Shapiro–Wilk and Kolmogorov–Smirnov tests were used to check normality assumption of linear mixed effect model. The histogram of the SBP measurement in Fig. 2a was identified to exhibit right skewed, and suggesting some transformation to meet the assumptions. To handle this problem logarithm transformation in Fig. 2c, d and square root transformation in Fig. 2b were considered. Thus, the square root transformation in Fig. 2b achieved normality (i.e., result from Shapiro–Wilk and Kolmogorov–Smirnov test is not significant, p-value = 0.452 and p-value = 0.068 respectively) implying that the square root transformed data set appear to follow a normal distribution.

**Figure 3:**
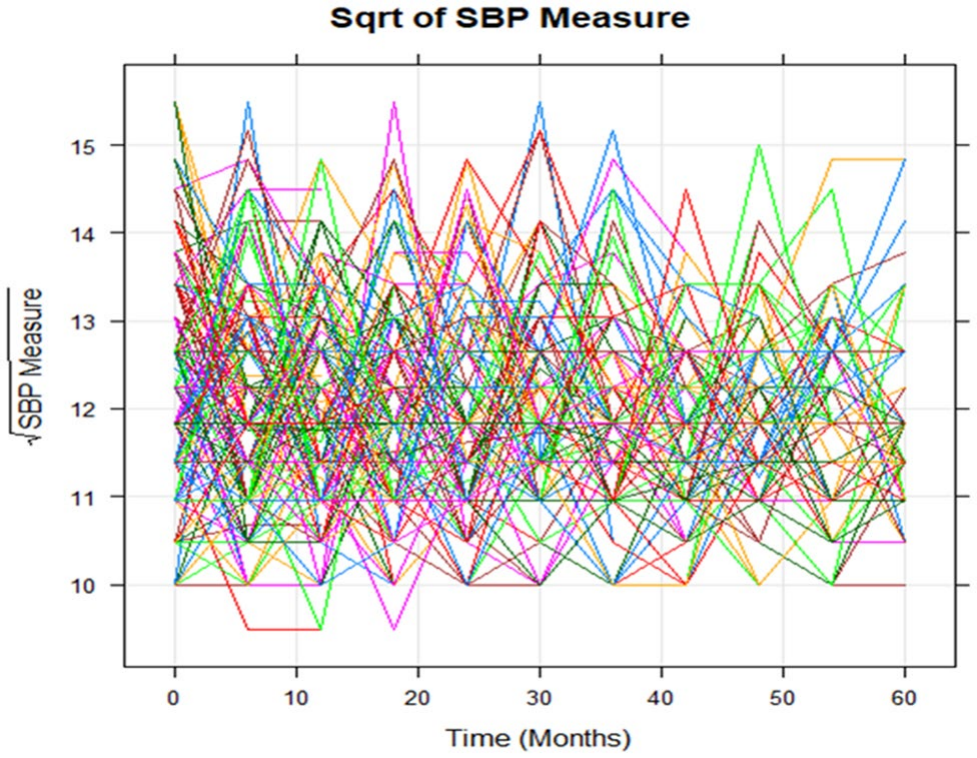
Plot of the individual profile of SBP of hypertension patients in follow up time.

Similarly, Fig. 3 Individual profile plot of SBP measurement of hypertension patients by follow up time and indicates some trajectories were steeper while others were almost horizontal, indicating the variability in SBP measurements overtime.

Figure 3 above showed individual profile plot of longitudinal SBP measurement eligible for the study. The plot provides information on variability between SBP measurement and shows there is change in SBP measurement over time. It may be inclining or decline over time. It appears that, there is a fluctuation in SBP over time and the variability of SBP measurement seemed larger at the beginning and lower at the end.

### Univariable analysis

To normalize SBP measurement*,* the square root of SBP measurement was used in the linear mixed effect models. We fitted univariable marginal models to explore the relationship between the square root of SBP measurement and each of the covariates. In this analysis we considered eleven variables. Among the, variables those which are significant at 25% modest level of significance in the univariable analysis were used as a candidate for the multivariable analysis.

The variables alcohol use, Khat intake, smoking tobacco, status of stress, stages of hypertension diseases, adherence to drug treatment, related diseases, family history of hypertension and fasting blood sugar were found to be significant at 25% level of significance and they are considered in the multivariable analysis.

### Multivariable analysis

In linear mixed effect model covariance structures should be carefully selected in order to obtain valid inferences for the parameters of fixed effects in the model^25^. From covariance structures, the one with the lowest AIC and BIC with convergence of the model in REML and ML were considered. In the analysis, four different commonly used covariance structures such as; Compound Symmetry (CS), Unstructured (UN), first Order Autoregressive (AR (1)) and Heterogeneous Compound Symmetry (HCS).

In Table 1 unstructured covariance (UN), indicated that AIC and BIC were smallest value, suggesting that the unstructured covariance (UN) covariance structure best fits our data.

**Table 1:** Analysis for the selection of the best fitting covariance structure.

Covariance structure	AIC	BIC	logLik
Compound symmetry (CS)	5951.32	6064.87	− 2955.66
Autoregressive (AR)	5941.61	6055.16	− 2950.80
Unstructured (UN)	5922.54	6011.53	− 2938.25
Homogeneous compound symmetry (HCS)	5940.28	6053.83	− 2950.14

After fixing the covariance structure with UN, It is an important to determine the random effect to be included in linear mixed effect model.

In Table 2 the computed result of AIC and BIC are indicated that random intercept model have lowered AIC and BIC values than random slope, and Random intercepts and slope model. Therefore, random intercept model were used in linear mixed effect model that appropriately predicts the mean change of the square root of SBP measurement over time.

**Table 2:** Selection of random effect models to be included in linear mixed effect model.

Models	AIC	BIC	LogLik
Random intercept model	5937.91	6040.10	− 2950.95
Random slope model	5941.62	6055.16	− 2950.80
Random intercept and slope model	5955.23	6062.16	− 2958.56

Table 3 show significant association of between the mean change in the square root of systolic blood pressure measurements, and the variables related diseases, adherence to treatment, stage of hypertension diseases, smoking tobacco, alcohol use, Khat intake and family history of at 5% level of significance. Being alcohol use significantly associated with in the mean change of SBP measurement. The mean change in the systolic blood pressure measurement was 0.51 times higher for alcohol used patients as compared to patients do not use alcohol. When we compare the two groups (Khat intakes, do not Khat intake) in term of their SBP measurements, we found that patients those had intake Khat have higher SBP measurement than patients do not intakes Khat. The mean change in the square root of SBP measurement of intake Khat was 0.47 times higher than those do not intake Khat controlling for the other variables. When we compare the two groups (Smoke tobacco, do not smoke tobacco) in terms of their SBP measurements, the patients who had smoke tobacco have higher SBP measurement than those who have not smoke tobacco. The mean change in SBP measurement of smoking tobacco was 0.158 times higher than those do not smoke tobacco keeping other variables constant. Adherence to treatment was associated with the mean change of SBP measurement and patients those had poor (low) adherence to treatment have higher SBP measurement compared to those have good (high) adherence to treatment. Mean change in SBP measurement of poor (low) adherence to treatment was 0.71 times higher than those who have good (high) adherence to treatment controlling for the other variables. When we compare the two groups (positive family history, negative family history) in terms of their SBP measurements, the patients those had positive family history of hypertension disease have higher SBP measurement compared to those had negative family history of hypertension disease. The mean change in SBP measurement of positive family history patients was 0.235 time higher compared to negative family history patients keeping constant other variables.

**Table 3:** Results of analysis of linear mixed effect model with random intercept.

Variables	Category	Estimate	Std.Error	t-value	p-value	95% CI
Intercept		11.710	0.146	79.714	0.001	[11.422, 11.998]
Observation time		− 0.001	0.001	− 0.691	0.489	[− 0.003, 0.002]
Alcohol use	No (ref)					
	Yes	0.51	0.062	8.20	0.0412	[0.072, 0.175]
Khat intake	No (ref)					
	Yes	0.47	0.068	6.87	0.0492	[0.087, 0.182]
Tobacco smoking	No (ref)					
	Yes	0.158	0.069	2.289	0.022	[0.023, 0.294]
Status of stress	No (ref)					
	Yes	0.100	0.071	1.418	0.156	[− 0.238, 0.382]
Stage of hypertension	Normal (ref)					
	Elevated	0.140	0.172	1.398	0.162	[− 0.096, 0.578]
	Stage 1	2.28	0.145	15.724	0.017	[0.057, 0.513]
	Stage 2	1.79	0.137	13.07	0.0191	[0.089, 0.448]
	Hypertensive crisis	0.479	0.148	3.236	0.001	[0.187, 0.772]
Adherence	Good (ref)					
	Poor	0.71	0.062	11.45	0.017	[0.391, 1.010]
Related disease	None (ref)					
	Stroke	0.81	0.094	8.617	0.008	[0.410, 1.210]
	Heart case	0.64	0.071	9.014	0.032	[0.350, 0.930]
	Other cases	0.72	0.114	6.316	0.025	[0.152, 0.297]
Family history	Negative (ref)					
	Positive	0.235	0.070	3.357	0.040	[0.002, 0.272]

Regarding stages of hypertension disease, average change in SBP measurement for hypertension patients those were in Elevated stage was 0.140 times higher compared to the normal stage. Also, the SBP measurements of patients at stage_1_, stage_2_ and hypertensive crisis stage were 2.28, 1.79 and 0.479 times higher compared to patients at the normal stage respectively; controlling for other variables. When we compare the two groups (patients having stroke case, Patients none stroke case) in terms of SBP measurements, the patients those having stroke related disease have higher mean change in SBP measurement compared to patients none related diseases. The mean change in SBP measurement was 0.81 times higher for patients having stroke cases compared to none cases. The average change in SBP measurement was 0.64 times higher for patients those having heart disease compared to patients none related diseases controlling for the other variables.

### Analysis of cox proportional hazard model

For each variable there is a univariable Cox proportional hazards model analysis that contains a single independent variable, which is used to know the significance of each variable with survival time. In the study, the variables that are significant in the univariable analysis in relation to time to the occurrence of event (or death) due to blood pressure were selected at 25% modest level of significance^26^. All potential variables that are supposed to have significant impact (p-value < 0.25) on the survival time of patients in univariable analysis were included in the multivariable cases.

### Assessment of proportional hazard (PH) assumptions

The proportional hazards assumption, which asserts that the hazard ratios are constant overtime and it, is important to use fitted PH model. Checking Cox PH model assumption is important to make inferences based on the models. The test of (PH) model assumption was done by applying formal test in the model by looking the estimated parameter over time. The regression coefficients were tested for each covariate and global test suggested strong evidence of non-proportionality (p < 0.025). The formal test for proportional hazard model was done. The analysis show that the time dependent covariates (interaction of covariates with logarithm of time) Khat (*χ*^2^ = 3.53, p < 0.066), related diseases (*χ*^2^ = 2.84, p < 0.0941), diabetes mellitus status (*χ*^2^ = 3.84, p < 0.054) and family history of hypertension disease (*χ*^2^ = 1.69, p < 0.198) were not statistically significant that means the proportional hazard assumption holds at 5% level of significance but the covariates age and adherence to drug treatment (*χ*^2^ = 15.85, p < 0.0002) and (*χ*^2^ = 7.29, p < 0.00967) respectively were statistically significant, therefore the proportional hazard assumption does not holds at 5% level of significance. Finally global test was significant at 5% level of significant. Therefore the Cox proportional hazard model assumption does not hold.

### Weibull regression model

In the analysis of survival data parameters are estimated using Weibull Regression model.

The Weibull model analysis in Table 4 below show that survival time of hypertension patients was significantly related with family history, stage of hypertension, related disease and smoking tobacco. Comparing hazard ratio (HR) by family history of hypertension, indicate that being positive family history of hypertension were 1.732 times higher risk of death than negative family history of hypertension (HR = 1.732, p-value = 0.045) and the risk of death for patients those smoke tobacco were 1.366 time higher than patients who did not smoke tobacco (HR = 1.336, p-value = 0.046).

**Table 4:** Results of analysis of survival data using Weibull model.

Variables	Category	Value	Std. Error	z-value	p-value	Exp ($\beta$)
Intercept		4.826	0.355	13.61	0.001	124.711
Family history	Negative (ref)					
	Positive	0.549	0.183	3	0.045	1.732
Stage of hypertension	Normal (ref)					
	Elevated	0.031	0.362	0.09	0.932	1.031
	Stage 1	0.286	0.332	0.86	0.390	1.331
	Stage 2	0.40	0.0299	13.377	0.0493	1.491
	Hypertensive crisis	0.164	0.0339	4.838	0.0428	1.578
Related disease	None (ref)					
	Stroke	0.185	0.0208	8.894	0.0375	1.203
	Heart case	0.437	0.198	2.20	0.028	1.548
	Other cases	0.221	0.0305	7.246	0.0469	1.247
Adherence	Good (ref)					
	Poor	0.011	0.145	0.07	0.942	1.011
Smoking tobacco	No (ref)					
	Yes	0.312	0.156	2.00	0.046	1.366

### Joint model analysis

After having appropriate separate models for the mean change of the square root of SBP measurement and time to death of patients due to hypertension, the next step is to explore an appropriate joint model that associates the longitudinally measured SBP and time to death of patients.

The analysis reveal that the association parameter ($\alpha$) was significant. Because of this, we employed the joint modeling approach following the variable in the Weibull survival model and linear mixed effect model.

In Table 5 comparing mean change in the square root of SBP measurement by status of stress, the mean change in square root of SBP was 0.165 times higher for hypertension patients those in stressed condition compared to patients not in stress conditions controlling for other independent variables. Regarding related diseases, the mean change in the square root of SBP measurement was 0.186 times higher for stroke case compared to none related case and heart case related disease was 0.193 times higher compared to none related disease of hypertension patients keeping constant for the other variables. The mean change in the square root of SBP measurement was 0.084 times higher for poor Adherence to treatment patients compared to good Adherence to treatment controlling for the other covariates.

**Table 5:** Longitudinal and survival sub-models with association parameter.

Variables	Category	Longitudinal sub-model			
		Estimate	Std.Error	t-value	p-value
Intercept		11.112	0.122	90.765	0.001
Observation time		− 0.002	0.002	− 0.994	0.365
Alcohol	No (ref)				
	Yes	0.064	0.0052	12.308	0.038
Khat intake	No (ref)				
	Yes	0.086	0.0048	17.917	0.043
Smoking tobacco	No (ref)				
	Yes	0.278	0.029	9.334	0.016
Status of stress	No (ref)				
	Yes	0.165	0.039	4.192	0.034
Stages of hypertension	Normal (ref)				
	Elevated	0.186	0.072	3.966	0.002
	Stage 1	0.187	0.0186	10.054	0.0154
	Stage 2	0.152	0.0086	17.674	0.046
	Hypertensive crisis	0.385	0.041	9.326	0.002
Adherence	Good (ref)				
	Poor	0.084	0.0043	19.535	0.0088
Related disease	None (ref)				
	Stroke	0.186	0.0096	19.35	0.0186
	Heart case	0.193	0.073	2.645	0.036
	Other cases	0.235	0.083	2.832	0.050
Family history	Negative (ref)				
	Positive	0.189	0.062	3.034	0.049
		**Survival sub-model**			
Family history	Negative (ref)				
	Positive	0.439	0.123	3.557	0.001
Stages of hypertension	Normal (ref)				
	Elevated	0.135	0.336	0.401	0.862
	Stage 1	0.298	0.248	1.201	0.042
	Stage 2	0.063	0.0186	3.387	0.025
	Hypertensive crisis	0.256	0.0255	10.039	0.032
Related disease	None (ref)				
	Stroke	0.155	0.0203	7.635	0.0376
	Heart case	0.533	0.098	5.405	0.032
	Other cases	0.286	0.0301	9.501	0.038
Adherence	Good (ref)				
	Poor	0.012	0.048	0.251	0.860
Smoking tobacco	No (ref)				
	Yes	0.356	0.135	2.629	0.044
Association ($\alpha$)		0.03	0.0038	7.895	0.034

The association parameter ($\alpha$) was significantly different from zero (p-value < 0.05), indicating that there is strong association between the square root of SBP measurement and the risk of death. The positive value of the association parameter (0.03) indicated that the slope of the square root of SBP measurement was positively associated with the risk of death, and with unit increase in the square root of SBP measurement the risk of death was increased.

## Discussion

The purpose of this study was to examine the association of survival time and systolic blood pressure measurement and the survival time of the hypertension patients. The current study reveals that family history, stage of hypertension, related diseases, adherence to treatment; and smoking tobacco were potential barriers that affect both survival time and blood pressure measurements of the hypertension patients. This study found that an age was associated with survival time of the patients. The finding results are similar with findings from a study in Uganda by Ref.^27^. However, this result was opposite to the research conducted in eastern Ethiopia by Ref.^28^.

We have also found a family history of hypertension was a statistically significant risk factor for death in hypertension patient; this result is in agreement with previous researchers^28,29^. Current study reveals that family history is significantly affect systolic blood pressure measurement. However, finding from other research in Jima University specialized Hospital contradictory to this study finding^30^.

The study reveals that Khat intake was highly associated with survival time of hypertension patients. This findings are similar with previous research finding by Refs.^31,32^ identified Khat intake is one potential risk factor of hypertension.

This study identified the association between smoking tobacco and systolic blood pressure measurements of hypertension patients, and smoking tobacco was significantly associated with an increased risk of hypertension. This finding is similar with previous research finding by Ref.^9^ identified smoking is one potential risk factor of hypertension. However, many researchers reported that there were no significant association between smoking and SBP of hypertension patients, and they concluded that there is no causal relationship^32,34^.The current study found that the patients who do not use alcohol had better survival probability than those who use alcohol. Similarly, Ayalew et al. indicated that Alcohol use is a potential risk factor of hypertension and they also found Hypertension was significantly higher in individuals who use alcohol than those who did not use^33^.

In this study the finding regards the relationship between place of residence of hypertension patient and the survival time until hypertension-related death was no significant association between them. So, the current study reveal that place of resident have no significant effect on SBP and survival time of the patients. But, many researchers reported that place of residence has a significant effect on hypertension patients^11,33^

Although this current study did not find any relationship between genders with patients a systolic blood pressure and the survival time until hypertension-related death; this finding is similar with previous research finding by Ref.^33^. But other studies in Ethiopia and India have reported that the covariate gender does not have any association with hypertension^35,36^.

The finding from the current study showed that related disease was significantly associated with both survival time and SBP measurements of hypertension patients. This finding was similar with previous researcher finding^37^. Similarly, stages of hypertension disease and adherence to treatment were potential barriers that associated with both survival time and SBP measurement of the patients in the current study. The finding was consistent with the previous study finding^38^. The study found that there is strong dependence between a systolic blood pressure and survival time of hypertension patients, and suggests that joint model analysis rather than separate model analysis for such kind of dependence outcomes, clinical and medical studies. These findings are agreed with previous researchers finding^12,13,23^.

## Conclusions

The study concludes that the covariates with significant effects on survival time and longitudinal systolic Blood Pressure are the family history, stage of hypertension diseases, related diseases, adherence to treatments, and smoking tobaccos were potential barriers that affect both survival time and blood pressure measurements of the patients. Also, the patients with good adherence, with normal blood cholesterol level, patients did not intakes Khat, and patients with a positive family history had better survival probability compare with patients with poor adherence, raised blood cholesterol level, patients intakes Khat, and patients with negative family history respectively.

The significance of the association parameter in the joint model indicates that there is a strong dependence of systolic blood pressure and survival time of hypertension patients. When evaluate the overall performance of the separate and the joint models, the joint model performs better for analysis of hypertension patient’s data.

In conclusion, there are significant numbers of hypertension patients were found in the study area, and great attention and intervention should be needed on identified potential barriers. Aggressive work by stakeholders towards awareness creation about the effect of potential barriers; promotion of healthy lifestyle and improving health checkups among the community, implementation of community-based screening programs needed for early detection of hypertension and to treat hypertension related diseases are highly recommended.
